# Validation of the Lung-Mol Graded Prognostic Assessment (GPA) System for the Prognosis of Patients Receiving Radiotherapy for Brain Metastasis From Non-small Cell Lung Cancer

**DOI:** 10.7759/cureus.57485

**Published:** 2024-04-02

**Authors:** Daichi Toriduka, Yukinori Matsuo, Hideki Hanazawa, Noriko Kishi, Megumi Uto, Takashi Mizowaki

**Affiliations:** 1 Department of Radiation Oncology and Image-Applied Therapy, Kyoto University Graduate School of Medicine, Kyoto, JPN; 2 Department of Radiation Oncology, Kindai University Faculty of Medicine, Osakasayama, JPN

**Keywords:** repeated courses of radiosurgery, survival prediction, radiotherapy, lung-molgpa, brain metastases

## Abstract

Purpose: The Lung-mol graded prognostic assessment (GPA) system predicts the prognosis of patients with brain metastases (BM) from non-small cell lung cancer (NSCLC) separately for adenocarcinoma and non-adenocarcinoma. This study aimed to validate the Lung-molGPA system using a cohort of patients in our institution who received radiotherapy for BM.

Materials and methods: Three hundred and thirty-nine patients with NSCLC who received their first course of radiotherapy for BM were included in the analysis. Among them, 65 received their second course of radiotherapy for BM. Data on sex, age, Karnofsky performance status (KPS), extracranial metastases (ECM), number of BM, histological type, and gene mutations were collected according to the Lung-molGPA system. We examined the validity of the scores assigned to the factors included in the Lung-molGPA system, separately for adenocarcinoma and non-adenocarcinoma. In addition, we validated the Lung-molGPA system to predict survival during both the first and second courses of radiotherapy.

Results: The factors in the Lung-molGPA were significantly associated with survival, except for age in non-adenocarcinoma with marginal significance. Regarding discrimination ability, the C-indices were 0.65 and 0.69 for adenocarcinoma and non-adenocarcinoma, respectively, in the first course of radiotherapy for BM, while those in the second course were 0.62 and 0.74, respectively. Survival prediction by Lung-molGPA was almost consistent with actual survival in the first course of radiotherapy, except for the score of 0-1.0 in both histologies and 2.5-3.0 in non-adenocarcinoma. In the second course of radiotherapy, median survival could be predicted for some patients with adenocarcinoma.

Conclusions: Our study confirms the validity of Lung-molGPA for the estimation of median survival based on patient characteristics at the time of initiation of radiotherapy for patients in the first course of radiotherapy and shows that it may be applicable to patients with adenocarcinoma in the second course of radiotherapy.

## Introduction

Brain metastases (BM) occur in 20%-40% of patients with cancer, leading to high rates of morbidity and mortality [1]. Half of all patients with non-small cell lung cancer (NSCLC) develop BM during their disease [2]. Epidermal growth factor receptor (EGFR) mutation and anaplastic lymphoma kinase (ALK) rearrangement are significantly associated with BM risk [3].

Brain metastases are treated by surgery, whole-brain radiotherapy (WBRT), stereotactic radiosurgery (SRS), stereotactic radiotherapy (SRT), chemotherapy, anti-angiogenic therapy, targeted therapy, or immunotherapy. The development of tyrosine kinase inhibitors (TKI) has improved the prognosis of patients with driver mutations such as EGFR and ALK rearrangements [3]. Whole-brain radiotherapy is the standard treatment for patients with multiple BM with high intracranial progression-free survival, but impairment of neurocognitive function is a crucial problem [4]. Consequently, SRS, or SRT, is being increasingly adopted as an alternative to WBRT to achieve good long-term local control while reducing the risk of neurocognitive deficiency [5]. In the US National Comprehensive Cancer Network (NCCN) guidelines (version 3, 2022), SRS or SRT is recommended for patients who have “limited” BM. Limited BM was defined in terms of the number of BM or total intracranial disease volume [6].

An accurate model of the prognosis of patients with BM from NSCLC is needed for the selection of appropriate treatment for individual patients. Prognostic scores such as recursive partitioning analysis (RPA) and graded prognostic assessment (GPA) have been used to predict the prognosis of patients with BM and determine treatment options [7, 8]. Furthermore, diagnosis-specific graded prognostic assessment (DS-GPA) provides a more accurate model specific to lung cancer [9]. Although the prognosis of patients with driver mutations has improved, the molecular features are not taken into consideration in these scores, and it is difficult to accurately predict the prognosis of patients with BM of NSCLC with EGFR mutations and ALK rearrangements. In 2017, a North American collaborative group advocated a scoring system to add molecular features (EGFR and ALK) to GPA, which is the Lung-molGPA system for NSCLC [10]. The Lung-molGPA system consists of five factors: age, Karnofsky performance status (KPS), extracranial metastases (ECM), number of BM, and gene status. This score can be used to more accurately predict the prognosis of patients with genetic mutations [11,12].

Because the prognosis of patients with BM has improved, some patients require multiple courses of radiotherapy [13,14]. However, no studies have validated the Lung-molGPA system in patients receiving a second course or further radiotherapy.

In this study, we validated the Lung-molGPA system for the prognosis of patients with BM of NSCLC who received a first and second course of brain radiotherapy.

## Materials and methods

This study was conducted in accordance with the principles of the Declaration of Helsinki. The institutional ethical review board of Kyoto University Graduate School and Faculty of Medicine, Kyoto, Japan, approved this study (approval number R1048-1).

Patients who underwent radiotherapy for BM of NSCLC at our institution between January 2007 and December 2016 were enrolled. Those who had a history of radiotherapy for BM or who did not complete radiotherapy for BM were excluded from the analysis.

Treatment for BM was determined based on the size and number of BM during the study period at our institution. Stereotactic radiosurgery or SRT is usually performed for patients with up to three BM and ≤3 cm, . For BM >3 cm, surgery was considered, or 10- to 13-fraction SRT was chosen considering the risk of radiation necrosis when the BM was inoperable. Whole-brain radiotherapy was performed in patients with meningeal dissemination or >4 BM. 

Validation of the factors in the Lung-molGPA system

First, we examined the validity of the scores assigned to the factors included in the Lung-molGPA system in our population, separately for adenocarcinoma and non-adenocarcinoma, during the first course of radiotherapy (Table 1). Data on the five factors for the Lung-molGPA system (age, KPS, ECM, number of BM, and gene status) and other characteristics (sex and histology) were collected for all patients. The number of BMs was defined as the number of metastases detected on diagnostic imaging performed before the start of each radiotherapy course. To reveal the impact of each factor on survival, we performed a multivariate Cox regression analysis.

**Table 1:** The Lung-molGPA system KPS: Karnofsky performance status; ECM: extracranial metastases; BM: brain metastases; EGFR: epidermal growth factor receptor; ALK: anaplastic lymphoma kinase

Factor	Criteria	Score
Age (in years)	≥ 70	0
	< 70	0.5
KPS	≤ 70	0
	80	0.5
	90-100	1
ECM	Present	0
	Absent	1
Number of BM	> 4	0
	1-4	0.5
Gene status	No or unknown	0
	EGFR mutation or ALK-positive	1

Validation of the Lung-molGPA system

We then validated the Lung-molGPA system to predict survival during both the first and second courses of radiotherapy. For each course of radiotherapy, the Lung-molGPA score was calculated based on the patient characteristics at the time of initiation of the radiotherapy course. We stratified the patient groups according to the Lung-molGPA system scores. The discriminatory ability of the Lung-molGPA system was evaluated using Harrell's concordance index (C-index). In addition, the agreement between the median overall survival (OS) predicted by Lung-molGPA and the observed OS was illustrated using a calibration plot. The median OS was estimated using the Kaplan-Meier method, measured from the first day of radiotherapy until death by any cause, and censored at the last follow-up date.

All statistical analyses were performed using EZR (Saitama Medical Center, Jichi Medical University, Saitama, Japan), a graphical user interface for R (R Foundation for Statistical Computing, Vienna, Austria, version 3.6.3) [15]; EZR is a modified version of R Commander version 2.6-2 that facilitates biostatistical evaluations.

## Results

**Figure 1:**
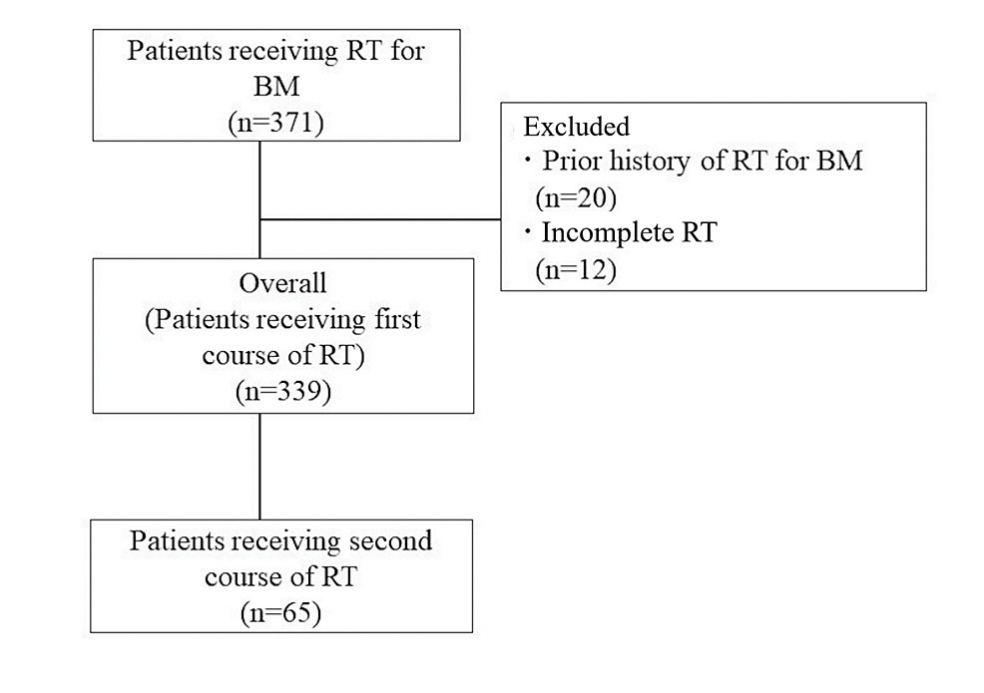
Patient flow diagram RT: radiotherapy; BM: brain metastases; n: number

Three hundred and seventy-one patients received radiotherapy for BM between January 2007 and December 2016. Among them, 32 patients were excluded because 20 had a history of radiotherapy for BM before this period, and 12 did not complete their radiotherapy treatment. In total, 339 patients who received their first course of radiotherapy were included in the analysis (Figure 1). Among them, 65 received their second course of radiotherapy for BM. The patient characteristics during the first and second courses of radiotherapy are shown in Table 2. 

**Table 2:** Patient characteristics KPS: Karnofsky performance status; ECM: extracranial metastases; BM: brain metastases; adeno: adenocarcinoma; non-adeno: non-adenocarcinoma; EGFR: epidermal growth factor receptor; ALK: anaplastic lymphoma kinase; n, number

Factor		Overall (first course) (n = 339)	Second course (n=65)
Sex	Female	144	23
	Male	195	42
Age (in years)	Median (range)	68 (36–89)	64 (37–79)
	≥ 70	142	25
	< 70	197	40
KPS	≤ 70	244	33
	80	44	22
	90-100	51	10
ECM	Present	237	47
	Absent	102	18
Number of BM	> 4	170	39
	1-4	169	26
Histology	Adeno	267	53
	Non-adeno	72	12
Gene status	No or unknown	208	40
	EGFR mutation or ALK-positive	131	25

Follow-up information regarding 339 patients was obtained from medical records in our institution or from information provided by hospitals to which patients were transferred. The data cut-off date was July 31, 2020. The median follow-up period was 7.2 months (range: 0.3-146.2 months). Details of the radiotherapy administered to the 339 patients are shown in Table 3. During the first course of radiotherapy, the most common type was WBRT (73.1%), followed by SRS or SRT (25.1%). In the second course, SRS or SRT (63.1%) was the most common, followed by WBRT (32.3%).

**Table 3:** Radiotherapy details RT: radiotherapy; WBRT: whole-brain radiotherapy; SRS: stereotactic radiosurgery; SRT: stereotactic radiotherapy; n: number; %: percent

Type of RT	Does regimen	First course (n = 339)	Second course (n = 65)
WBRT	30 Gy/10 fr.	196 (57.8 %)	17 (26.1 %)
	37.5 Gy/15 fr.	15 (4.4 %)	2 (3.1 %)
	other	37 (10.9%)	2 (3.1 %)
SRS	20 Gy/1 fr.	43 (12.7 %)	15 (23.1 %)
	others	7 (2.1 %)	-
SRT	20–28 Gy/5 fr.	23 (6.8 %)	21 (32.3 %)
	39 Gy/13 fr.	3 (0.9 %)	3 (4.6 %)
	other	9 (2.6 %)	2 (3.1 %)
Gamma knife	other	6 (1.8 %)	3 (4.6 %)

Validation of the factors in the Lung-molGPA system

**Figure 2:**
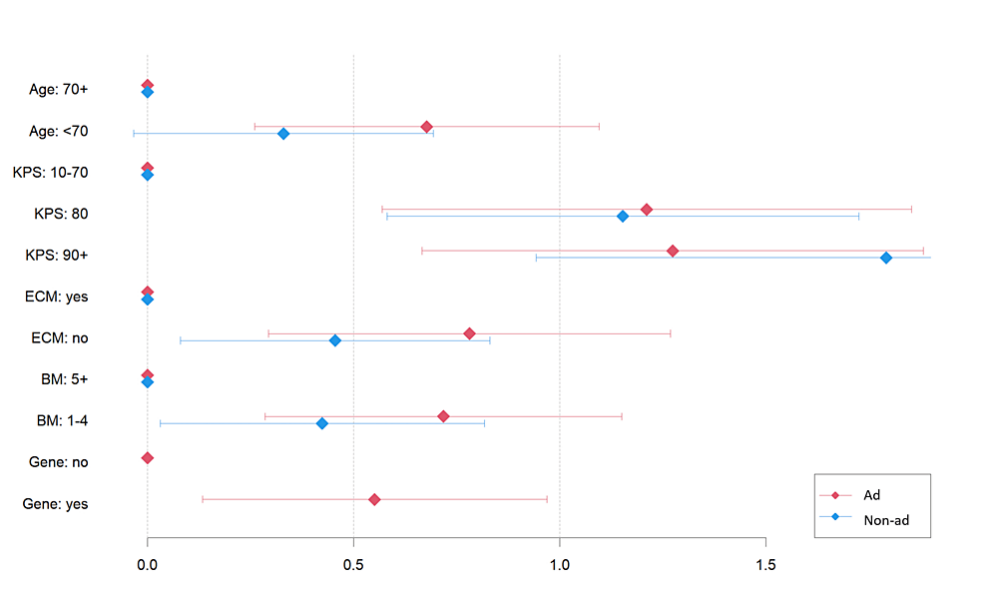
Coefficients (β) in the Cox regression model for the factors used in the Lung-molGPA model; the coefficients were normalized with the sum of the values equal to that of the Lung-molGPA scores. KPS: Karnofsky performance status; ECM: extracranial metastases; BM: brain metastases; gene: gene mutation; ad: adenocarcinoma; non-ad: non-adenocarcinoma

In adenocarcinoma, age <70 years, KPS = 80, KPS = 90-100, absence of ECM, the presence of one to four BM, and gene mutations were significantly associated with a decreased risk of death for the first course of radiotherapy (Table 4). In non-adenocarcinoma patients, all factors except age were significantly associated with OS. The relative impact of KPS = 80 seems higher than that of Lung-molGPA, while the relative impact of ECM absence in non-adenocarcinoma and gene mutation in adenocarcinoma seems lower than that in Lung-molGPA (Figure 2).

**Table 4:** Multivariate analysis of prognostic factors for overall survival for the first radiotherapy KPS: Karnofsky performance status; ECM: extracranial metastases; BM: brain metastases; EGFR: epidermal growth factor receptor; ALK: anaplastic lymphoma kinase; adeno: adenocarcinoma; non-adeno: non-adenocarcinoma; coef: coefficient; HR: hazard ratio; %: percent; CI: confidence interval; n: number; reference: reference value; NA: not available

Factor	Categories		Adeno (n = 267)					Non-adeno (n =72)	
		Coef	HR (95% CI)	P-value			Coef	HR (95% CI)	P-value
Age	≥ 70	0	1		Reference		0	1	Reference
	< 70	-0.45	0.64 (0.49–0.84)		0.001		-0.50	0.60 (0.35–1.05)	0.075
KPS	≤ 70	0	1		Reference		0	1	Reference
	80	-0.80	0.45 (0.30–0.69)		< 0.001		-1.77	0.17 (0.07–0.41)	< 0.001
	90-100	-0.84	0.43 (0.29–0.65)		< 0.001		-2.74	0.06 (0.02–0.24)	< 0.001
ECM	Present	0	1		Reference		0	1	Reference
	Absent	-0.51	0.60 (0.43–0.82)		0.002		-0.70	0.50 (0.28–0.88)	0.017
Number of BM	>4 brain metastases	0	1		Reference		0	1	Reference
	1-4 brain metastases	-0.47	0.62 (0.47–0.83)		0.001		-0.65	0.52 (0.29–0.95)	0.035
Gene mutation	No or unknown	0	1		Reference		NA	NA	NA
	EGFR mutation or ALK-positive	-0.36	0.70 (0.53–0.92)		0.010		NA	NA	NA

Validation of the Lung-molGPA system in the first course of radiotherapy

**Figure 3:**
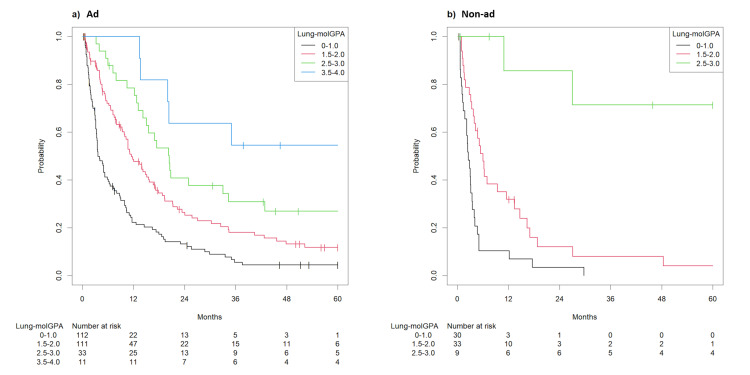
Survival after the first course of radiotherapy for brain metastasis according to the Lung-molGPA system ad: adenocarcinoma; non-ad: non-adenocarcinoma; Lung-molGPA: Lung-mol graded prognostic assessment (a) Kaplan-Meier curve showing survival by the Lung-molGPA system after the first course of radiotherapy for adenocarcinoma; (b) Kaplan-Meier curve showing survival by the Lung-molGPA system after the first course of radiotherapy for non-adenocarcinoma

For lung adenocarcinoma, most patients had unfavorable prognostic features, and their scores were 0-1.0 in 112 patients (41.9%) and 1.5-2.0 in 111 patients (41.5%). Thirty-three patients (12.3%) had scores of 2.5-3.0, while those remaining had scores of 3.5-4.0. In lung adenocarcinoma, cases with scores of 0-1.0, 1.5-2.0, 2.5-3.0, and 3.5-4.0 had median OS of 3.7 (95% confidence interval (CI): 3.4-6.1), 11.5 (95% CI: 10.0-16.7), 20.4 (95% CI: 15.1-42.9), and 79.4 months (95% CI: 20.3-not available (NA)), respectively (p<0.001, Figure 3). Its C-index was 0.65 (95% CI: 0.62-0.69). In patients with non-adenocarcinoma, scores were 0-1.0 in 30 patients (41.7%) and 1.5-2.0 in 33 patients (45.8%). Nine patients (12.5%) had scores of 2.5-3.0. The corresponding median OS values were 2.6 (95% CI: 2.2-3.5), 6.0 (95% CI: 4.0-13.4), and 72.0 months (95% CI: 27.0-NA) (p<0.001, Figure 3). Its C-index was 0.69 (95% CI: 0.63-0.76).

**Figure 4:**
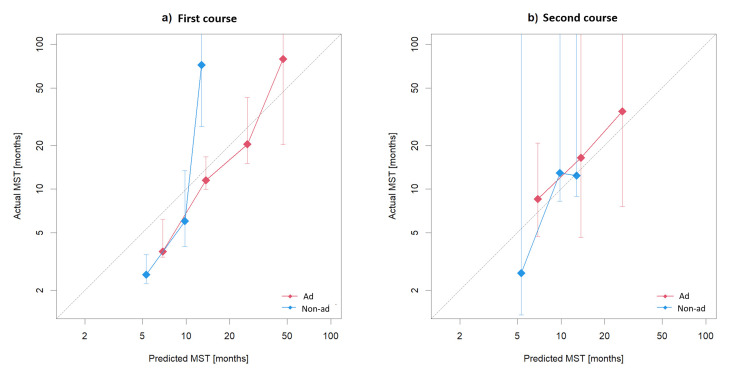
Calibration plot for survival after the first (a) and second (b) course of radiotherapy ad: adenocarcinoma; non-ad: non-adenocarcinoma; MST: median survival time (a) Calibration plot for survival after the first course of radiotherapy for adenocarcinoma and non-adenocarcinoma; (b) Calibration plot for survival after the second course of radiotherapy for adenocarcinoma and non-adenocarcinoma

Survival prediction by Lung-molGPA was almost consistent with actual survival in the first course of radiotherapy, except for the score of 0-1.0 in both histologies and 2.5-3.0 in patients with non-adenocarcinoma (Figure 4a).

Validation of the Lung-molGPA system in the second course of radiotherapy 

**Figure 5:**
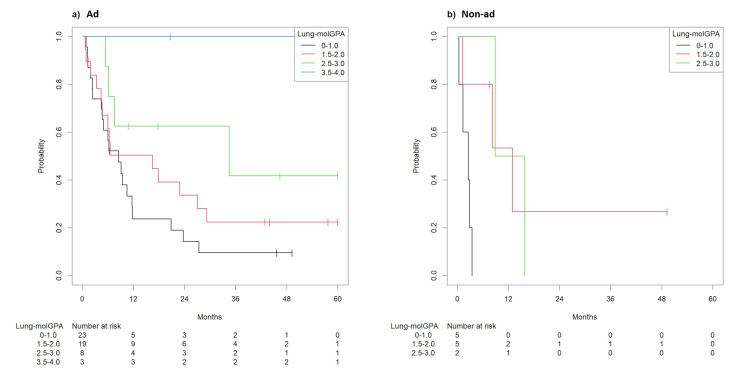
Survival after the second course of radiotherapy for brain metastasis according to the Lung-molGPA system ad: adenocarcinoma; non-ad: non-adenocarcinoma; Lung-molGPA: Lung-mol graded prognostic assessment (a) Kaplan-Meier Curve showing survival by the Lung-mol GPA system after the second course of radiotherapy for adenocarcinoma; (b) Kaplan-Meier Curve showing survival by the Lung-mol GPA system after the second course of radiotherapy for non-adenocarcinoma

In lung adenocarcinoma, most patients who received a second course of radiotherapy were classified into the poor prognosis group. The scores were 0-1.0 in 23 patients (43.4%) and 1.5-2.0 in 19 patients (35.8%). Eight patients (15.1%) had scores of 2.5-3.0, and the remaining three (5.7%) had scores of 3.5-4.0. Cases with scores of 0-1.0, 1.5-2.0, 2.5-3.0, and 3.5-4.0 had median OS of 8.5 (95% CI: 4.7-20.8), 16.5 (95% CI: 4.6-NA), and 34.5 (95% CI: 7.6-NA) months and not reached, respectively (p = 0.034, Figure 5). Its C-index was 0.62 (95% CI: 0.54-0.71).

In non-adenocarcinoma, scores of 0-1.0 were in five patients (41.6%) and 1.5-2.0 in five patients (41.6%). Two patients (16.8%) had scores of 2.5-3.0. The corresponding median OS values were 2.6 (95% CI: 1.4-NA), 12.9 (95% CI: 8.3-NA), and 12.4 months (95% CI: 8.9-NA). Its C-index was 0.74 (95% CI: 0.61-0.86). They revealed the difference between the groups (p = 0.022), but the stratification order of the original model was not preserved (Figure 4b).

## Discussion

We performed a retrospective validation study of Lung-molGPA in a population of Japanese patients. Several studies have validated the Lung-molGPA system in different countries and races; however, it is not clear how many times the patients received radiotherapy and who received other initial treatments such as surgery [10-12]. To the best of our knowledge, this is the first study to validate the Lung-molGPA system from the first and second courses of radiotherapy for BM in lung cancer based on the patient characteristics at the time of initiation of each radiotherapy course.

The prognosis of lung cancer patients with BM has improved with the development of new treatment methods, and the number of patients who receive repeated radiotherapy for BM is increasing [13,14]. However, it is also important to identify patients with poor prognoses for the selection of appropriate treatment and to assist them in making better treatment choices. A randomized clinical trial showed that WBRT cannot improve quality of life or OS more than optimal supportive care, including dexamethasone, for patients with a poor prognosis [16]. The Cox proportional hazard model analysis of the first-course cohort confirmed that the five factors included in Lung-molGPA were significantly associated with survival, except for age, in patients with non-adenocarcinoma with marginal significance. In adenocarcinoma, the coefficients of gene status and KPS deviated from the Lung-molGPA system scores. In non-adenocarcinoma, KPS and the number of BM had a higher impact on OS than the original Lung-molGPA system. Potential explanations for these results are as follows: First, in this study, the presence of genetic mutations was unknown in 57 of 267 cases (21.0%), including some cases from the old era, in which genetic mutation estimation was inadequate. The high incidence of a lack of mutation information might affect the divergence in gene mutation scores from those obtained with the original Lung-molGPA system. In addition, the status of resistance to TKI was not considered for scoring at the time of the second radiotherapy. Second, there was an uneven distribution of KPS in our study. Karnofsky performance status is a subjective index and may differ among evaluators due to clinician bias, which may be related to the divergence in the specific weights of KPS compared to the original system [17]. Third, the difference in the imaging modalities used to detect BM between our report and the original report may be related to the divergence in the specific weights of the number of BM compared to the original system.

In the first course of radiotherapy, patients with both histologies had inferior survival scores (0-1.0) to those in the original report (adenocarcinoma, median 3.7 vs. 6.9 months; non-adenocarcinoma, median 2.6 vs. 5.3 months). Conversely, non-adenocarcinoma patients with a score of 2.5-3.0 had better survival rates compared with those in the original report (median 72.0 vs. 12.8 months). Survival in the other stratifications was generally consistent with that in the original report. The potential explanations for these results are as follows: First, in the population of the original report, unlike in our study, surgery was performed in 13% of patients, and we believe that the difference in treatment modality was related to the difference in survival rate. Second, in patients with non-adenocarcinoma with a score of 2.5-3.0, four out of nine patients (44.4%) received SRS as the first course of radiotherapy, while three received SRS as the second. According to Shultz et al., repeated SRS after an initial course of SRS yields high rates of local, favorable durations of overall and neurological survival [13]. We believe that Lung-molGPA may be able to identify a group of patients who can achieve long-term survival with a repeated course of SRS. In the second course of radiotherapy, the stratified order of the original model was preserved, and patient survival in adenocarcinoma was generally consistent in each stratification compared to that in the original report (median 8.5 vs. 6.9 months; median 16.5 vs. 13.7 months; median 34.5 vs. 26.5 months; median NA vs. 46.8 months) [10]. However, the stratified order of the original model was not maintained for non-adenocarcinoma, and the validity of the original report was not confirmed. The sample size of non-adenocarcinoma patients who underwent second radiotherapy was not sufficient, and we believe that further cases are needed to validate the Lung-mol GPA for patients receiving a second course of radiotherapy.

This study has some limitations. First, the study design was retrospective, and the database of a single institution was analyzed. Compared with the original report of a North American collaborative group, our study had a limited number of patients [10]. Second, only patients who underwent radiotherapy for BM were included in this study. Other factors that influence the local control of BM and OS, such as surgery, chemotherapy, targeted drugs, and immunotherapy, were not considered in this study. In addition, the inclusion of only patients who have undergone radiotherapy may be a cause of selection bias. Third, the study was conducted primarily on a group of patients before the advent of immune checkpoint inhibitors, and the expression of programmed death ligand-1 (PD-L1) was not estimated. In the updated version of Lung-molGPA, the expression of PD-L1 was considered [18]. Fourth, while some patients received more than two courses of radiotherapy for recurrent BM, our study was limited to patients who received a first and second course only. Further studies are needed to evaluate the applicability of our results to patients who receive more than two courses of radiotherapy. Finally, the period of patient selection is old in this study. For a thorough evaluation of a prognostic model, we need a number of patients with long-term follow-up. Therefore, we have chosen the time frame for patient selection to ensure an adequate observation period in this study.

## Conclusions

Our study confirms the validity of Lung-molGPA for the estimation of median survival based on patient characteristics at the time of initiation of radiotherapy for patients in the first course of radiotherapy and shows that it may be applicable to patients with adenocarcinoma in the second course of radiotherapy. Additional studies are needed to confirm the validity of the Lung-molGPA system and to consider the influence of systemic therapy and repeated radiotherapy on survival.
